# Efficacy evaluation of a minimally invasive surgical procedure (oblique lateral interbody fusion) for lumbar spinal tuberculosis—retrospective cohort study

**DOI:** 10.3389/fbioe.2024.1500234

**Published:** 2024-12-17

**Authors:** Ke Zheng, Zhihao Ni, Guosong Han, Tao Shan, Bin Xu

**Affiliations:** ^1^ Department of Orthopaedics, The First Affiliated Hospital of Anhui Medical University, Hefei, Anhui, China; ^2^ Department of Spine Surgery, The First People’s Hospital of Hefei City, Hefei, Anhui, China

**Keywords:** oblique lateral interbody fusion, posterior percutaneous pedicle screw fixation, biomechanic, debridement, spinal tubercolosis

## Abstract

**Objective:**

In the current study, to demonstrate the advantages of oblique lateral interbody fusion (OLIF), we focused on the therapeutics for lumbar spinal tuberculosis with the comparison of three treatments, including anterior approach, posterior approach, and OLIF combined with posterior percutaneous pedicle screw fixation.

**Methods:**

This study included patients with lumbar spinal tuberculosis from July 2015 to June 2018. We divided these patients into three groups: 35 patients underwent an anterior-only approach (Group A), 36 patients underwent a posterior-only approach (Group B), and 31 patients underwent OLIF combined with posterior percutaneous pedicle screw fixation (Group C). Operation time, blood loss, hospital stays, the visual analog scale (VAS) and the Oswestry disability index (ODI), ASIA grade, the erythrocyte sedimentation rate (ESR), C-reactive protein (CRP), and Cobb angle were used to evaluate the surgical approaches.

**Results:**

A total of 102 patients joined this study of three therapeutic groups. The mean hospital stays, the mean operative time, and surgical blood loss of the three groups of patients were (14.40 ± 2.6, 14.00 ± 2.51, and 9.39 ± 1.86) days, (177.23 ± 13.23, 198.00 ± 16.75, and 150.39 ± 14.28) minutes, and (307.43 ± 21.91, 406.67 ± 27.02, and 105.97 ± 18.90) mL, respectively. VAS and ODI of all patients significantly improved 1 week after surgery (*P* < 0.05). As all patients received regular anti-tuberculosis treatment before and after surgery, ESR and CRP indicators maintained at normal levels 1 week after surgery. The Cobb angle was significantly corrected 1 week after surgery (*P* < 0.05). Eight patients had postoperative complications, and all of them recovered after active treatment.

**Conclusion:**

OLIF combined with posterior percutaneous pedicle screw fixation has the advantages of less surgical trauma and faster postoperative recovery, although all three surgical approaches can achieve satisfactory clinical results.

## 1 Introduction

*Mycobacterium tuberculosis*, which stains positive for the acid-fast stain, is the pathogenic bacterium that causes tuberculosis (TB). TB remains a global public health threat despite the emergence of new diagnostics, drugs, and therapeutic options (Churchyard et al., 2017). TB consists of two main types of infections: pulmonary tuberculosis and extrapulmonary tuberculosis (EPTB). EPTB affects areas of the body other than the lung, such as bones, spine, or joints, which is called bone or skeletal TB, reducing the patient’s quality of life. Spinal TB is one of the most common forms of skeletal TB, with the thoracolumbar spine most commonly affected (Du et al., 2022; Khanna and Sabharwal, 2019). Spinal TB often leads to complications such as paravertebral abscess, bone destruction, and spinal instability (spinal deformity and vertebral collapse) (Jain and Kumar, 2013; Zhuang et al., 2021). Anti-tuberculous chemotherapy is currently considered the best treatment for milder forms of spinal TB with no neurological deficit. In contrast, surgical treatment is the best option for patients with neurological complications or vertebral instability and spinal deformity (Louw et al., 2020; Dunn and Ben Husien, 2018).

Debridement of infected bone and other tissue is an important part of the surgical treatment of TB. In patients with spinal tuberculosis, debridement has also been shown to play an important role in controlling abscess formation (Dunn and Ben Husien, 2018; Du et al., 2020; Fuentes Ferrer et al., 2012). There are two main surgical strategies for spinal tuberculosis: anterior and posterior. Anterior surgery provides direct access to the intervertebral disc, has the advantage of debridement under direct vision, and may increase the fusion rate of the surgical wound (Li et al., 2019). The large surgical trauma in anterior surgeries also brings the risk of damaging the iliac blood vessels, peritoneal contents, ureters, and autonomic nervous system (Sasso et al., 2005). On the other hand, posterior surgery has good deformity correction and stable reconstruction capabilities, which is the most significant advantage (Wu et al., 2022). Posterior surgery is an indirect debridement method. Compared with anterior surgery, it is not particularly thorough in removing abscesses caused by tuberculosis. This surgical method would not only increase the probability of late recurrence but also increase the risk of *M. tuberculosis* being introduced from the anterior column to the posterior column (Ukunda and Lukhele, 2018). Furthermore, posterior surgery may cause chronic low back pain or lower limb weakness, which may be caused by unilateral or bilateral dissection of the facet joint and opening of the spinal canal during the operation. In general, both anterior and posterior surgeries have advantages and disadvantages, and the choice between them remains controversial (Ekinci et al., 2015). However, reducing trauma is necessary to improve the effectiveness of treating spinal tuberculosis. Novel surgical methods are urgently needed.

With the advancement of surgical methods and materials technology, minimally invasive surgery provides new options for the treatment of spinal tuberculosis. Minimally invasive surgery techniques have gained wider acceptance among surgeons performing lumbar diseases because they allow direct access and visualization of intervertebral discs in order to achieve a more complete discectomy and theoretically a better fusion, while potentially decreasing morbidity (Eck et al., 2007; Shen et al., 2007; Kaiser et al., 2002). Oblique lateral interbody fusion (OLIF) is a minimally invasive technique of lumbar anterior approach (Mayer, 1997)and officially named “OLIF” by Silvestre et al., in 2012 Silvestre et al. (2012). Specifically, the psoas muscle is pushed back in OLIF to allow surgical passage through the space between the aorta and psoas muscle to reach the target vertebrae or intervertebral space. Using OLIF reduces blood loss, speeds recovery, and reduces the likelihood of nerve damage. Moreover, the surgical approach of OLIF has shown definite clinical results in the treatment of degenerative lumbar diseases (Jin et al., 2018; Woods et al., 2017; Ye et al., 2020; Shimizu et al., 2021). Additionally, from a biomechanical perspective, tuberculosis lesions extensively damage the intervertebral discs and vertebral bodies, leading to disc space collapse. This in turn causes local kyphotic deformities, significantly disrupting the sagittal plane balance of the spine (Tobing et al., 2021). Through sufficient interbody fusion and posterior supplemental fixation, OLIF can better correct sagittal plane imbalance of the spine (Xiao et al., 2020). We speculate that OLIF would have great potential in treating lumbar tuberculosis.

In the current study, we used OLIF combined with posterior percutaneous pedicle screw fixation to treat spinal tuberculosis. Compared with traditional anterior and posterior surgical methods, our combined treatment took advantage of minimally invasive technology and achieved more satisfactory clinical results. This study provides evidence of evidence-based medicine for the clinical application of OLIF technology.

## 2 Methods

### 2.1 Inclusion and exclusion criteria

#### 2.1.1 Inclusion criteria

(1) From 18 to 70 years of age; (2) Diagnosis of lumbar tuberculosis was confirmed by clinical and radiographic examinations; (3) Antituberculosis drugs were administered for at least 4 weeks.

#### 2.1.2 Exclusion criteria

(1) Tuberculosis involvement of multiple lumbar spine levels; (2) Erythrocyte sedimentation rate (ESR) or C-reactive protein (CRP) did not stabilize or decrease after 4 weeks of antituberculosis drugs; (3) Could not receive follow-up for at least 1 year; (4) A history of nervous system or mental illness; (5) Patients with active pulmonary tuberculosis, spinal tumors, or acute vertebral fractures of the thoracic and lumbar spine.

### 2.2 Subjects

We retrospectively included patients who suffered from lumbar spinal tuberculosis and had undergone surgical treatment between July 2015 and June 2018. A total of 102 patients in two Grade-A tertiary hospitals were included in this study and divided into three groups: 35 patients underwent anterior-only approach (anterior-only group, Group A), 36 patients underwent posterior-only approach (posterior-only group, Group B), and 31 patients underwent OLIF combined with posterior pedicle fixation (Group C). Preoperative diagnosis was consistent among the three groups, and there was no significant difference in general physical condition and disease course. The study protocol was approved by the Medical Ethical Committee. All patients have obtained informed consent.

### 2.3 Surgical procedures

**FIGURE 1:**
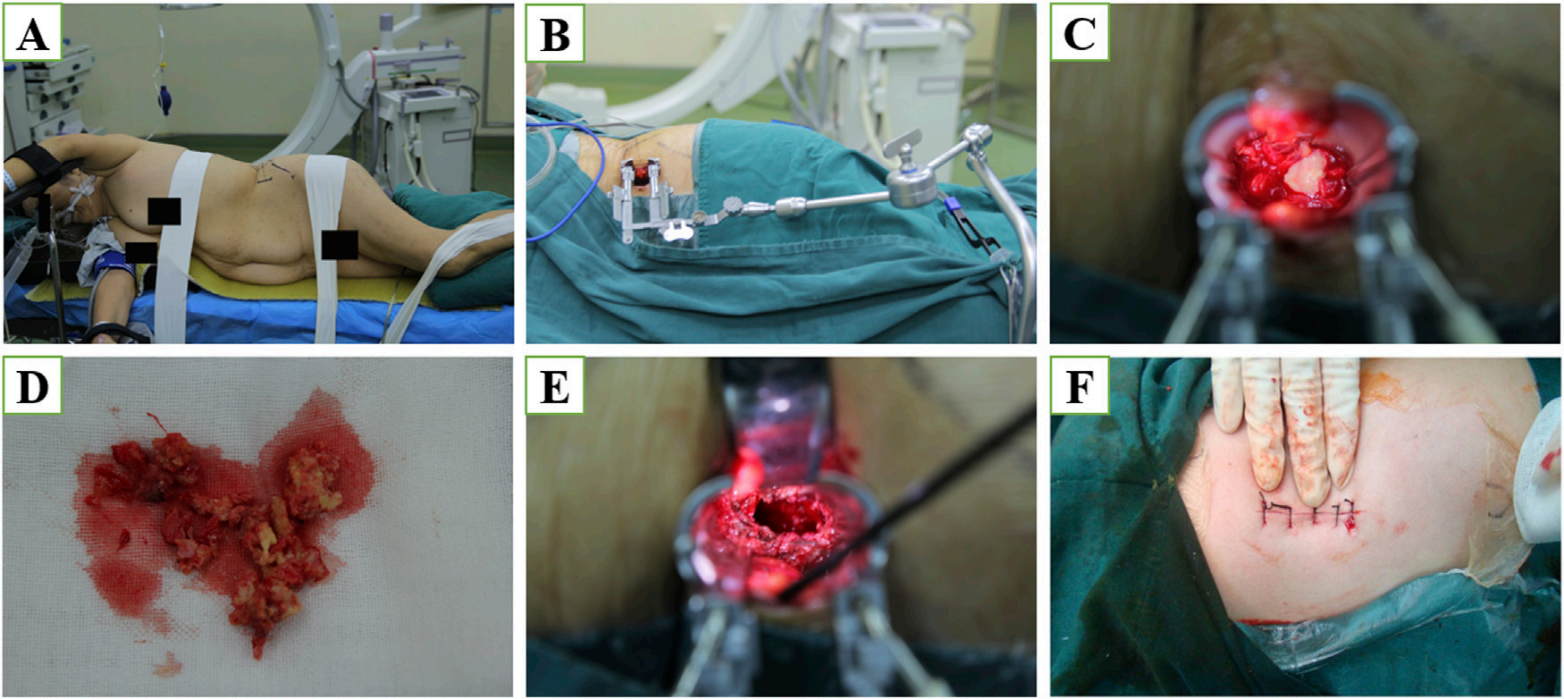
**(A)** Skin marking to check the disk level using a C-arm X-ray imager. A longitudinal skin incision was made 5–7 cm anterior to the front of the centrum (a minimum of 5 cm is recommended). The peritoneum was cleared from the psoas using blunt dissection. **(B)** Sequential dilation was used to place a working channel. **(C)** Abscess secondary to lumbar spinal tuberculosis. **(D)** Dead bone and a small amount of abscess tissue were removed. **(E)** The remaining unaffected vertebral body was used as a graft bed. **(F)** The width of the incision is only three fingers.

OLIF group: Once general anesthesia was applied, the patient was placed in the lateral position and a C-arm machine was used to identify the vertebral segments to be debrided. After routine sterilization, an incision of approximately 5 cm in length was made in the lateral abdominal region. The external abdominal oblique, internal abdominal oblique and transversus abdominis muscles were sequentially exposed and separated layer by layer. The extraperitoneal fat was then carefully pushed away using the fingers to access the retroperitoneal space and move the peritoneal contents forward to locate the anterior edge of the psoas muscle with the periosteum, and then an S-shaped pull-hook was placed to protect the large abdominal vessels. The anterior edge of the vertebral body was explored and the infected tissue was excised. The abscess, granulation tissue, caseous necrotic material, and necrotic intervertebral discs were then all removed. Then, a drain was placed and the incision was closed layer by layer. The patient was adjusted to the prone position and posterior internal fixation was performed using percutaneous pedicle screw instrumentation. c-arm machine was checked to confirm that the screws were in good position, and finally, the wound was irrigated and closed layer by layer. The procedure of the surgery is illustrated in Figure 1.

### 2.4 Evaluation of operative outcomes and complications

We classified and compiled the basic information of the included patients, including their age, gender, and body mass index, respectively. The intraoperative indexes, including operative time and blood loss, were prospectively recorded. The kinds and numbers of complications were carefully recorded, including neurologic injury, instrument failure, lower extremity numbness and weakness, and incisional infection. The length of hospital stays was also documented.

### 2.5 Evaluation of follow-up indexes

#### 2.5.1 General outcome

Outcome measures were examined preoperatively and at one, six, twelve and 18 months (final follow-up) after surgery. Radiographs, CT, and MRI were performed preoperatively and postoperatively at each follow-up. At the final follow-up interbody fusion status was graded by the Brantigan, Steffee, Fraser (BSF) scale. The double-blind method was used to read and evaluate intervertebral bony fusion by two professional orthopedic surgeons. Other outcomes examined were CRP, ESR level, and Oswestry Disability Index (ODI) score.

#### 2.5.2 American spinal injury association (ASIA) grade

Neurologic function was assessed by American Spinal Injury Association (ASIA) grade before surgery and at final follow-up. Briefly, A = Complete loss: No sensory or motor function is preserved in sacral segments S4-S5; B = Incomplete: Sensory, but not motor function is preserved below the neurologic level and extends through sacral segments S4-S5; C = Incomplete: Motor function is preserved below the neurologic level, and most key muscles below the neurologic level have a muscle grade of less than III; D = Incomplete: Motor function is preserved below the neurologic level, and most key muscles below the neurologic level have a muscle grade that is greater than or equal to III; E = Normal: Sensory and motor functions are normal.

#### 2.5.3 Visual analog scale (VAS)

Back pain changes were evaluated using a visual analog scale (VAS). A score of 0 indicates no pain, while 10 means the worst pain, subjectively.

### 2.6 Statistical method

Variables were represented as mean ± standard deviation (SD). Changes in VAS, ODI, and CRP at 18 months follow up were tested using paired *t*-test. All statistical assessments are two-sided. *P*-value <0.05 was considered statistically significant. SPSS version 23.0 software was used for data analysis (IBM, Armonk, NY, United States).

### 2.7 Statement

This work has been reported in line with the STROCSS criteria (Tobing et al., 2021). This research and was registered in China Clinical Trial Registry.

## 3 Results

### 3.1 General results

We analyzed the basic information of all patients included in the study. There were no differences in age, gender and body mass index among patients who underwent three different surgical methods. Before surgery, all patients received adequate preoperative anti-tuberculosis treatment preparation. There was no significant difference in preoperative clinical data examination (*P* > 0.05, Table 1). However, there were significant differences in intraoperative and postoperative indicators of the three surgical methods (anterior, posterior and OLIF). As shown in Table 2, the mean hospital stays, mean operation time and operative blood loss of patients in the three groups were (14.40 ± 2.6, 14.00 ± 2.51, and 9.39 ± 1.86) days, (177.23 ± 13.23, 198.00 ± 16.75, and 150.39 ± 14.28) minutes, and (307.43 ± 21.91, 406.67 ± 27.02, and 105.97 ± 18.90) mL. This highlighted that OLIF can reduce operation time and blood loss, as well as reduced the patient’s hospital stays.

**TABLE 1:** Baseline characteristics of the enrolled patients.

Factors	A (n = 35)	B (n = 36)	C (n = 31)	P-value
Age, y	48.49 ± 8.77	50.25 ± 8.97	48.90 ± 7.39	0.657
Gender, male/female	17/8	14/12	14/7	0.632
BMI (kg/m^2^)	19.70 ± 2.47	20.35 ± 2.49	20.32 ± 2.49	0.472
Preoperative VAS	6.51 ± 1.17	6.58 ± 1.03	6.48 ± 1.12	0.930
Preoperative ODI (%)	62.51 ± 15.97	62.81 ± 13.40	62.61 ± 14.39	0.995
Preoperative ASIA grade, A-C/D-E	5/30	5/31	5/26	0.964
Duration of neurologic symptoms, m	1.91 ± 2.72	1.75 ± 2.31	1.71 ± 2.61	0.940
Preoperative ESR (mm/h)	67.00 ± 26.32	66.75 ± 26.03	68.97 ± 23.85	0.929
Preoperative CRP (mg/L)	54.93 ± 23.08	54.05 ± 30.47	59.10 ± 27.32	0.726
Preoperative Cobb angle (^o^)	21.26 ± 5.64	19.56 ± 5.64	19.26 ± 5.78	0.298

^a^ anterior.

^b^ posterior.

^c^ OLIF.

**TABLE 2:** Comparison of major results after surgery in the three groups.

Group	Hospital stay, days	Operation time, mins	Blood loss, mL	VAS		ODI (%)		ASIA grade A-C/D-E Last follow-up	ESR (mm/h)		CRP (mg/L)		Cobb angle (^o^)		Postoperative complications		
				P	L	P	L		P	L	P	L	P	L	Incision infection	Instrument failure	Lower limb weakness and numbness
A	14.40 ± 2.65	177.23 ± 13.23	307.43 ± 21.91	5.00 ± 0.84^†^	1.11 ± 0.72^†‡^	62.51 ± 15.97	15.26 ± 12.40^†‡^	1/34	29.69 ± 5.37^†^	7.57 ± 4.09^†‡^	19.86 ± 2.64^†^	4.76 ± 2.94^†‡^	13.71 ± 1.89^†^	14.40 ± 1.77^†^	2	0	1
B	14.00 ± 2.51	198.00 ± 16.75^a^	406.67 ± 27.02^a^	4.89 ± 0.62^†^	1.19 ± 0.67^†‡^	63.75 ± 4.00	15.89 ± 12.05^†‡^	1/35	30.31 ± 5.62^†^	6.92 ± 4.66^†‡^	18.81 ± 3.23^†^	4.44 ± 2.10^†‡^	10.36 ± 1.52^a†^	10.72 ± 1.32^a†^	1	1	2
C	9.39 ± 1.86^a,b^	150.39 ± 14.28^a,b^	105.97 ± 18.90^a,b^	2.65 ± 0.61^a,b†^	1.16 ± 0.64^†‡^	44.61 ± 4.81^a,b†^	15.97 ± 9.14^†‡^	0/31	31.16 ± 5.94^†^	7.32 ± 4.64^†‡^	19.40 ± 2.90^†^	4.41 ± 2.54^†‡^	10.77 ± 1.52^a†^	11.10 ± 1.49^a†^	0	0	1
P-value^*^	<0.05	<0.05	<0.05	<0.05	>0.05	<0.05	>0.05	>0.05	>0.05	>0.05	>0.05	>0.05	<0.05	<0.05			

*Compared to each groups; † Compared to preoperative, P < 0.05. ‡ Compared to Postoperative 1 week, P< 0.05.

^a^P < 0.05 compared with group A.

^b^P < 0.05 compared with group B; P, Postoperative 1 week. L, last follow up.

**FIGURE 2:**
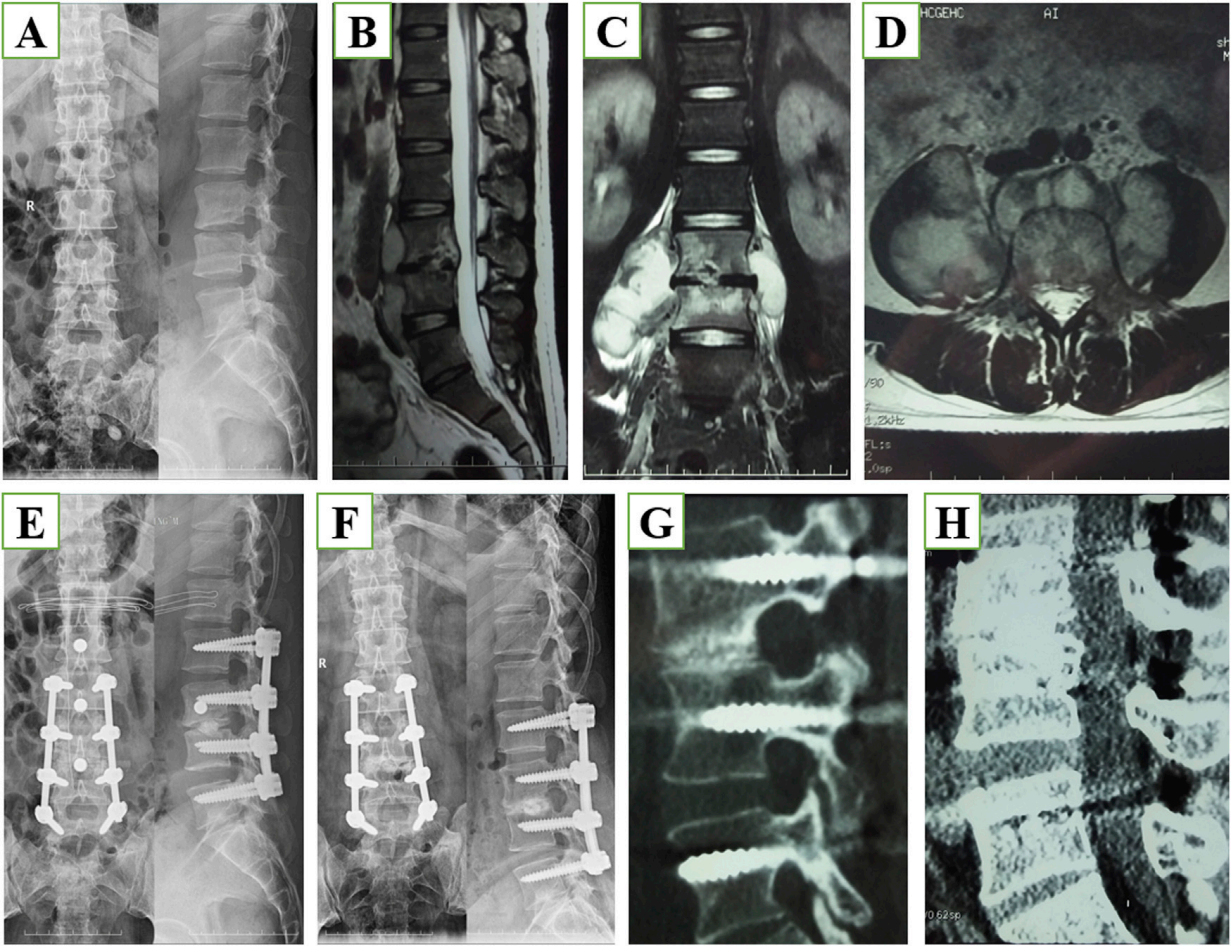
**(A)** Anterior-posterior and lateral x-ray images before surgery. **(B)** Magnetic resonance imaging (MRI), sagittal section, of the lumbar spine before surgery. **(C)** Lumbar MRI coronal section before surgery. **(D)** Lumbar MRI axial section before surgery. **(E)** X-ray images immediately after surgery. **(F)** X-ray images 10 months after surgery. **(G)** Computed tomography (CT) using the bone window 10 months after surgery. **(H)** CT using soft tissue window 10 months after surgery.

We analyzed the preoperative and postoperative imaging of the included patients, as shown in Figures 2A–D, which show the preoperative X-ray images of lumbar spine tuberculosis patients in frontal and lateral positions, and the sagittal, coronal, and axial views of MRI. Through the images we can observe that lumbar tuberculosis invaded the vertebral body and formed an abscess cavity in the paravertebral area. Figures 2E–H shows the postoperative X-ray, and postoperative 10-month X-ray and CT images. We can observe that in October postoperatively the patient had good internal fixation in place and fusion between the vertebrae was accomplished, which was confirmed by the CT images.

### 3.2 C-reactive protein and erythrocyte sedimentation rate

We evaluated the efficacy of surgical treatments by detecting patients’ CRP and ESR levels. Before the operation, we tested all patients included. The CRP and ESR levels of the patients in the anterior, posterior and OLIF groups were (67.00 ± 26.32, 66.75 ± 26.03, 68.97 ± 23.85) mm/h and (54.93 ± 23.08, 54.05 ± 30.47, and 59.10 ± 27.32) mg/L, with no significant difference between the three groups (*P* > 0.05).

After surgery, we measured the ESR and CRP to evaluate the tuberculosis lesion clearance rate and monitored the recurrence 1 week after surgery and at the last follow-up. We all implemented formal anti-tuberculosis standard postoperative treatment for the three groups of patients. As shown in Table 2, the levels of ESR and CRP 1 week after surgery were significantly lower than those before surgery. However, there was no difference between the three groups after surgery, indicating that the three surgical methods plus regular anti-tuberculosis treatment can improve patients’ conditions.

At the final follow-up, the levels of ESR and CRP in the three groups have improved significantly compared with 1 week after surgery, but there is no difference between the three groups after surgery (*P* > 0.05). This is consistent with surgical removal of lesions and the standard postoperative treatment of anti-tuberculosis.

### 3.3 Visual analog scale (VAS)

We used VAS to evaluate the pain relief of postoperative patients in different surgical groups. The preoperative VAS of the three groups were 6.51 ± 1.17, 6.58 ± 1.03, and 6.48 ± 1.12, respectively, with no significant difference (*P* > 0.05). However, the VAS of the three groups after surgery were 5.00 ± 0.84, 4.89 ± 0.62, and 2.65 ± 0.61. The VAS of the three groups showed a decreasing trend 1 week after surgery. Moreover, compared with the other two groups, the VAS of the patients in Group C were lower (*P* < 0.05). This indicated that the patient’s pain can be relieved after surgery, especially with OLIF surgery.

At the final follow-up, VAS of different groups showed a downward trend compared with 1 week after surgery, but there was no difference between the three groups at this time (*P* > 0.05). This suggested that all three surgical methods can relieve the patient’s pain at the final follow-up, and postoperative care and regular treatment play an important role in this.

### 3.4 Oswestry Disability Index (ODI)

According to the ODI, the patients’ neurological deficits have also been improved. The preoperative ODI of the three groups were 62.51 ± 15.97, 62.81 ± 13.40, and 62.61 ± 14.39, respectively, with no difference between the three groups (*P* > 0.05). However, the ODI of the three groups 1 week after surgery were 62.51 ± 15.97, 63.75 ± 4.00, and 44.61 ± 4.81. The VAS of groups B and C showed a decreasing trend 1 week after surgery, but the ODI of Group A did not decrease significantly. Importantly, compared with the other two groups, the VAS of the OLIF surgery group (Group C) was lower 1 week after surgery (*P* < 0.05). This OLIF surgery can improve the patient’s functional deficits in the short term. However, at the last follow-up, there was no difference in ODI among the three postoperative groups (*P* > 0.05), indicating that all three surgical treatments can restore the patient’s function, while OLIF performed better in the short term.

### 3.5 Cobb angle

According to the preoperative testing, we found that the preoperative Cobb angles in the three groups of patients were 21.26 ± 5.64, 19.56 ± 5.64, and 19.26 ± 5.78, respectively. There was no difference among the three groups (Table 1) (*P* > 0.05). However, during the follow-up 1 week after surgery, the Cobb angles of the three groups of patients were 13.71 ± 1.89, 10.36 ± 1.52, and 10.77 ± 1.52. Compared with Group A, the Cobb angles of groups B and C had a more significantly downward trend (*P* < 0.05). At the last follow-up, the Cobb angles among the three groups were 14.40 ± 1.77, 10.72 ± 1.32, and 11.10 ± 1.49. The results at the last follow-up were consistent with the results 1 week after surgery. Surgery can improve the patient’s scoliosis, and compared with the surgery in Group A, the surgical effect in groups B and C is more significant.

### 3.6 Complications

Complications occurred intraoperatively and postoperatively in all the three surgical methods. The number of complications in groups A, B and C were 3, 4, and 1, respectively. The case of complications in Group C was one patient with lower limb numbness and weakness, the number of which appeared in groups A and B were 1 and 2, respectively. Regarding wound infection, 2 cases occurred in Group A and 1 case occurred in Group B, but none occurred in Group C. This is closely related to the surgical method of OLIF, which is a minimally invasive surgical method with minimum risk of surgical infection. For the above complications, we took specific measures to help patients recover, including debridement dressings, neurotrophic treatment and rehabilitation training, etc. These complications have all been resolved.

## 4 Discussion

Bone and joint tuberculosis consist of a group of serious infectious diseases associated with the human skeleton (bones and/or joints), the incidence of which has increased, especially in underdeveloped countries, partly due to the increase of immunocompromised population. Tuberculous spinal infections (i.e., spinal tuberculosis) are common forms of bone and joint tuberculosis, accounting for nearly 50% of tuberculous osteoarticular manifestations (Pigrau-Serrallach and Rodriguez-Pardo, 2013). Of note, the lower thoracic and upper lumbar spine are the most frequently involved sites in spinal tuberculosis. Severe infections lead to instability of the vertebral body, deformity, and even neurological defects and paraplegia. Therefore, clinical treatments for spinal tuberculosis aim to eradicate the infection, prevent or decrease neurological deficits, and correct and avoid the development of spinal deformity (Khanna and Sabharwal, 2019). Although chemical drugs (isoniazid, rifampicin, streptomycin, or pyrazinamide) are the cornerstone of anti-tuberculous therapy (Rajasekaran and Khandelwal, 2013; Moon et al., 2002), they cannot completely clear vertebral infections and paravertebral abscesses and establish spinal stability. Surgical treatments are essential to coordinate with anti-tuberculous chemotherapy. Our findings in the current study suggested that OLIF is a feasible single-level spinal tuberculosis treatment option compared with traditional anterior or posterior approaches.

OLIF has been used to treat degenerative lumbar diseases, and in our opinion, it has its own advantages during surgery and in postoperative recovery in the treatment of lumbar TB. For the surgical treatment of lumbar tuberculosis, the traditional anterior or posterior approach exposes the lesion directly to the field of view, which makes the surgery traumatic and destructive. In contrast, the most intuitive advantage of the OLIF procedure used in this experiment is the smaller operating window during the procedure, in which we make a smaller skin incision centered on the vertebral segment for exposure (Silvestre et al., 2012), for muscle detachment, we dissect the external oblique, internal oblique and transversus abdominis muscles, etc., along the direction of their fibers, and bluntly detach them by accessing to the retroperitoneal space, thus realizing the removal of the lesion. The OLIF procedure in this experiment is a minimally invasive approach, which can greatly avoid muscle disarticulation and tissue damage, and reduce the probability of nerve and vascular damage. In the traditional posterior approach, the above-mentioned steps are performed in a blind field, especially when the lesion is located in the paravertebral region, in which case a surgical window must be created by removing the vertebral plate and articular eminence on one side, and even though pedicle screws can be performed directly under the surgical field to perform fixation, such an open surgery undoubtedly increases the surgical time and the amount of bleeding. For the traditional anterior approach, the larger surgical scope and its muscle soft tissues, etc. are important influencing factors that increase the amount of bleeding and operation time, which also directly lengthens the patient’s hospitalization time (*P* < 0.05). The operative time, bleeding, and hospitalization time is an intuitive way to evaluate the trauma of a surgical procedure. Thus, in our present study, the operative time, bleeding, and hospitalization time of the OLIF procedure used for the treatment of lumbar spinal tuberculosis were lower than those of traditional anterior or posterior procedures (*P* < 0.05), which is inextricably linked to the smaller incision exposure of the procedure. For the OLIF surgical approach to spinal tuberculosis, the anterior minimally invasive incision and muscle stripping reduced patient bleeding, accessed multiple levels through minimally invasive incisions, removed lesions under direct vision, and reduced the incidence of abdominal wall pain. Internal fixation is then performed through posterior percutaneous screws, which avoids exposure of open posterior wounds and incision of the lamina, among other things (Alander and Cui, 2018). Importantly, posterior percutaneous pedicle screw fixation has been widely used in recent years for the treatment of degenerative spinal disorders and fractures with satisfactory outcomes (Tian et al., 2018; Jin et al., 2020). The posterior approach of percutaneous pedicle fixation reduces complications such as chronic back pain or lower limb weakness caused by traditional posterior surgery, which may be due to the stripping of unilateral or bilateral paravertebral muscles and the opening of the spinal canal during the surgical procedure (Epter et al., 2009; Lee et al., 2017). Moreover, OLIF combined with posterior pedicle screw fixation provides optimal biomechanical stability (Guo et al., 2020). This surgical approach to the treatment of spinal tuberculosis allows early control and timely fusion, thereby correcting spinal deformity, enabling patients to get out of bed earlier, and reducing postoperative risks. Therefore, the number of cases of postoperative infections occurring after OLIF surgery was zero compared to the other two surgical approaches in our study.

VAS and ODI scores are another important indicator of patients’ postoperative recovery. Through the evaluation of patients’ postoperative pain, we found that the patients’ VAS and ODI scores 1-week post-surgery of OLIF surgical method were lower than those of traditional anterior and posterior surgical methods (*P* < 0.05), which is closely related to the surgical method. We considered that this might be related to less trauma in OLIF surgery, less pain in the short term after surgery and faster recovery of motor function of the body. However, at the final follow-up, there was no difference in the VAS and ODI scores of the three surgical methods (*P* > 0.05). The reason might be that, over time, all patients reached a state of bone graft fusion and achieve good results in recovery. There was no significant difference in the indicators of ESR and C-reactive protein between the three surgical methods during the follow-up period (*P* > 0.05). This might be mainly due to the fact that standardized antibiotics were implemented after all three surgical methods. In terms of correction of spinal deformity, we found that compared with pure anterior surgery or posterior surgery, OLIF can improve the Cobb angle (*P* < 0.05), and the Cobb angle is also statistically significant at follow-up. This also highlights that restoring intervertebral height through OLIF can better improve lumbar lordosis, facilitating the recovery of biomechanical stability (Guo et al., 2020).

However, our study has certain limitations. We only evaluated the combined treatments for spinal tuberculosis. The current study is mainly retrospective, limited by the small number of patients and short follow-up time.

## Data Availability

Publicly available datasets were analyzed in this study. This data can be found here: https://www.clinicaltrials.gov/ChiCTR2400079815.
